# 

**Published:** 1843-07

**Authors:** 


### CONTENTS

### OF NO. I.

### ORIGINAL COMMUNICATIONS.

### ESSAYS AND CASES.

Art. I.—A Monograph on the Nature and Treatment of Artificial Anus. By Samuel D. Gross, M.D., Professor of Surgery in the Louisville Medical Institute. 1

### REVIEWS.

Art. II.—Mémoire sur les Rétrécissements Organiques du Canal de l'Urètre et sur l'Emploi de Nouveaux Instruments de Sacrification, &c.

Memoir on Organic Strictures of the Urethra and on the Employment of new Scarifying Instruments for the Radical Cure of that Disease; with plates. By Martial Dupierris, M.D., of New-Orleans; 8vo: Paris, 1840. - - . - - - 48

### BIBLIOGRAPHICAL NOTICES.

Art. III.—Treatise on the Dental Art, founded on Actual Experience.—Illustrated by two hundred and forty-one figures in Lithography, and fifty-four Wood-cuts. By F. Maury, Dentist of the Royal Polytechnic School. Translated from the French, with notes and additions, by J. B. Savier, Doctor of Dental Surgery. Philadelphia: Lea and Blanchard, 1842. p. 324. - 55

Art. IV.—A Practical Treatise on the Management and Diseases of Children. By Richard T. Evanson, M.D., and Henry Maunsell, M.D., second American, from the fourth Dublin edition, with notes, by D. F. Condie, M.D. Philadelphia: Barrington and Haswell, 1843. - - - - - - 55

SELECTIONS FROM AMERICAN AND FOREIGN JOURNALS.

Observations on some Popular Remedies of German Practitioners. - - - - - - - - 57
Delirium Tremens treated by Ammonia. - - - 66
Advantages of Cultivating Medical Physiognomy. - - 66
Strangulated Hernia treated by Exhaustion through the Elastic Tube. - - - - - - - 68
Effusion into the Brain. - - - - - - 70
Liquor Taraxaci. - - - - - - - 71
Assafœtida in Hooping-Cough. - - - - 71

ORIGINAL INTELLIGENCE.

Travelling Editorials. - - - - - 73
Medical Society of Tennessee. - . - - - 78
Medical Society of Nashville. - - - - - 79
New York Journal of Medicine and the Collateral Sciences. 80
Lectures on Visceral and Surgical Anatomy, and Operative Surgery. - - - - - - 80

